# Low preoperative serum fibrinogen level is associated with postoperative acute kidney injury in patients with in acute aortic dissection

**DOI:** 10.1186/s13019-023-02114-7

**Published:** 2023-01-07

**Authors:** Xin-Liang Guan, Lei Li, Wen-Jian Jiang, Ming Gong, Hai-Yang Li, Yu-Yong Liu, Xiao-Long Wang, Hong-Jia Zhang

**Affiliations:** Beijing Laboratory for Cardiovascular Precision Medicine, Department of Cardiac Surgery, Beijing Aortic Disease Center, Beijing Institute of Heart Lung and Blood Vessel Diseases, Beijing Engineering Research Center of Vascular Prostheses, Beijing Anzhen Hospital, Capital Medical University, No.2 Anzhen Street, Beijing, 100029 China

**Keywords:** Acute kidney injury, Fibrinogen, Body mass index, Risk factors, Acute aortic dissection

## Abstract

**Objective:**

Acute kidney injury (AKI) after cardiac surgery is associated with serious complication and high risk of mortality. The relationship between hemostatic system and the prognosis of patients with acute type A aortic dissection (ATAAD) has not been evaluated. The purpose of this study was to investigate the association between preoperative serum fibrinogen level and risk of postoperative AKI in patients with ATAAD.

**Methods:**

A total of 172 consecutive patients undergoing urgent aortic arch surgery for ATAAD between April 2020 and December 2021 were identified from Beijing Anzhen Hospital aortic surgery database. The primary outcome was postoperative AKI as defined by the Kidney Disease Improving Global Outcomes (KDIGO) criteria. The univariate and multivariate logistic regression analysis were done to assess the independent predictors of risk for postoperative AKI. Receiver operating characteristic (ROC) curve was generated to evaluate the predictive probabilities of risk factors for AKI.

**Results:**

In our study, 51.2% (88/172) patients developed postoperative AKI. Multivariate logistic regression analysis identified low preoperative serum fibrinogen level (OR, 1.492; 95% CI, 1.023 to 2.476; *p* = 0.021) and increased body mass index (BMI) (OR, 1.153; 95% CI, 1.003 to 1.327; *p* = 0.046) as independent predictors of postoperative AKI in patients with ATAAD. A mixed effect analysis of variance modeling revealed that obese patients with low preoperative serum fibrinogen level had higher incidence of postoperative AKI (*p* = 0.04). The ROC curve indicated that low preoperative serum fibrinogen level was a significant predictor of AKI [area under the curve (AUC), 0.771; *p* < 0.001].

**Conclusions:**

Low preoperative serum fibrinogen level and obesity were associated with the risk of postoperative AKI in patients with ATAAD. These data suggested that low preoperative serum fibrinogen level was preferred marker for predicting the postoperative AKI, especially in obese patients with ATAAD.

## Introduction

Acute kidney injury (AKI) is an adverse postoperative complication of cardiac surgery which is independently associated with a longer hospital stay and higher risk of mortality [1–3]. Despite improved perioperative management and surgical techniques, the patients with acute type A aortic dissection (ATAAD) who underwent urgent total arch replacement (TAR) combined with a frozen elephant trunk (FET) implant generally still had an high risk of postoperative AKI [3–5]. Although there were some well-defined and effective strategies for both prevention and treatment of AKI, postoperative AKI was still recognized as a frequent and serious complication in aortic surgery. Therefore, early recognizing and identification for relevant risk factors of AKI preoperatively in patients with ATAAD provides an opportunity for surgeon to optimize high risk patients and to initiate preventative and therapies.

As the final substrate in the coagulation cascade and the ligand of platelet glycoprotein IIb/IIIa receptors, fibrinogen is conventionally used as the standard replacement in many European countries for managing postoperative bleeding [6, 7]. However, fibrinogen is also related to other medical conditions, including systemic inflammation and AKI [8]. For these reasons, we conducted a retrospective cohort study to investigate the relationship between preoperative serum fibrinogen level and AKI in patients with ATAAD using a multivariate logistic regression model containing all known associated major perioperative predictors.

## Methods

### Patient population

**Fig. 1:**
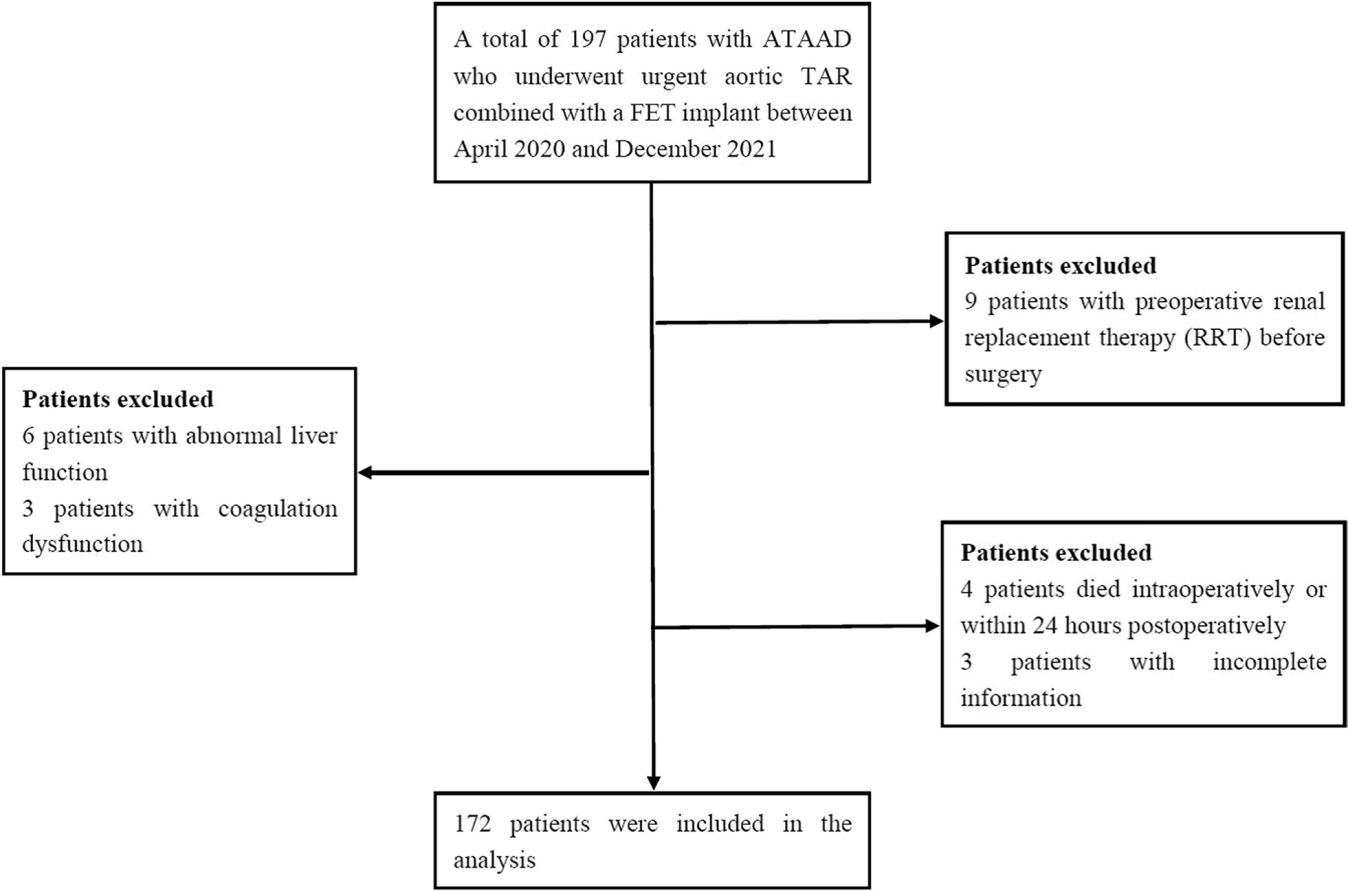
Flow diagram of the screening and enrollment of study patients

A total of 172 patients with ATAAD from Beijing Anzhen Hospital aortic surgery database who underwent urgent aortic TAR combined with a FET implant with cardiopulmonary bypass (CPB) between April 2020 and December 2021 were included in this analysis. Exclusion criteria included preoperative renal replacement therapy (RRT), incomplete clinical data and the conditions that impact fibrinogen level expressions (e.g., liver function disorder or coagulation/blood dysfunction). Patients who died intraoperatively or within 24 h postoperatively were also excluded because no meaningful data were available for the evaluation of postoperative AKI (Fig. 1). The study was approved by the institutional review board and the need for individual patient consent waived.

### Study design

In this single-center retrospective study, we analyzed the preoperative characteristics, operative details and postoperative outcomes of 172 consecutive patients (124 men and 48 women; age range, 27–75 years; average age, 48.4 ± 5.7 years) with ATAAD underwent TAR combined with a FET implant at Beijing Anzhen Hospital. The primary endpoint of this study was to evaluate the incidence and risk factors of postoperative AKI. ATAAD was diagnosed by enhanced computed tomography scan and aortic valve regurgitation was confirmed by echocardiography. Renal malperfusion was diagnosed as at least one renal artery dissection with creatinine rise above 50% of the normal upper limit [9]. The postoperative AKI was defined based on the Kidney Disease Improving Global Outcomes (KDIGO) criteria [10, 11]. All procedures were performed by the same surgery team. Trained staff collected detailed data from recruited patients from the electronic medical records at our medical center.

### Surgical procedures

Standard anesthetic management was used with endotracheal intubation. The procedure refers to total arch replacement using a tetra-furcate vascular graft in combination with the implantation of a special stented graft into the descending aorta. Briefly, the procedure is performed with right axillary artery cannulation for CPB and antegrade cerebral perfusion [5–15 mL/(kg·min)] under moderate hypothermic circulatory arrest (HCA). After systemic heparinization (300 U/kg bodyweight and maintaining an activated clotting time longer than 480 s), CPB was established. During CPB, temperature-adjusted flow rates of 2.5 L/(min·m^2^) were used, and the mean arterial pressure was generally maintained between 50 and 70 mmHg. Our policy was to completely excise the primary tear according to the extent of disruption in each case. This procedure involves the implantation of a FET into the descending aorta, TAR with a 4-branched vascular graft, and a specific sequence for aortic reconstruction (proximal descending aorta, then left carotid artery, ascending aorta, left subclavian artery, and finally innominate artery). After completing distal anastomosis, CPB was reinstituted, and the patient was gradually rewarmed to a normal temperature after a 5-min period of cold reperfusion for free radical washout. Proximal anastomosis was then performed.

### Statistical analysis

The normality of the data distribution was tested using the Kolmogorov–Smirnov test. Data are expressed as the mean ± standard deviation (SD) for continuous data with a normal distribution, as the median (25th percentile and 75th percentile) for continuous data with a nonnormal distribution, or numbers and percentages for categorical values. For comparison, one-way analysis or the Wilcoxon rank sum test was used for continuous variables, and the chi-square test or Fisher’s exact test was used for categorical variables. Logistic regression models were used to identify univariate and multivariate predictors for postoperative AKI. Univariate logistic regression analysis was used first to identify possible risk factors for postoperative AKI, and the multivariate model included variables that were found significant in the univariate analysis. In addition, to evaluate the effects of body mass index (BMI) and preoperative serum fibrinogen level for postoperative AKI, we created a mixed-effect analysis of variance model. The receiver operating characteristic (ROC) curve was generated to evaluate the predictive probabilities of risk factors for AKI using the area under the curve (AUC), 95% confidence interval (CI), specificity, and sensitivity. The optimal cutoff value was determined by calculating the Youden index of the ROC curve. For all analyses, a probability value of less than 0.05 was considered statistically significant. All statistical analyses were performed using SPSS 18.0 (SPSS, Inc., Chicago, IL).

## Results

### Baseline characteristics

After the exclusion criteria were applied, the total study cohort consisted of 172 consecutive patients with a mean age of 48.4 ± 5.7 years (range 27–75 years) in this study. The demographic, preoperative, intraoperative, and postoperative clinical data associated with AKI development were summarized in Table 1. Of these patients, there 124 were male and 48 were female. As shown in Table 1, the majority of patients with ATAAD had chest pain (93.9%) as the predominant preoperative symptom. Hypertension was present in 131 of the 172 patients. The median preoperative serum creatinine (sCr) was 79.5 (64.9, 99.9) umol/L and average estimated glomerular filtration rate (eGFR) was 86.3 ± 16.7 mL/(min•1.73m^2^). The median preoperative serum fibrinogen level for all patients was 3.0 (2.2, 4.1) g/L.

**Table 1:** Characteristics of the study patients with ATAAD at baseline

Characteristics	Non-AKI (*n* = 84)	AKI (*n* = 88)	*p* value
*Demographic data*			
Age, year	47.9 ± 10.9	48.8 ± 10.7	0.71
Male, %	62 (73.8)	62 (70.5)	0.73
BMI, kg/m^2^	25.0 ± 4.1	28.1 ± 3.2	0.009
*Medical history*			
Hypertension, %	64 (76.2)	67 (76.1)	0.96
Diabetes mellitus, %	2 (2.4)	4 (4.5)	0.59
Cerebrovascular disease, %	6 (7.1)	4 (4.5)	0.61
Smoking history, %	32 (38.1)	48 (54.5)	0.13
Marfan syndrome, %	6 (7.1)	7 (8.0)	0.51
*Preoperative condition*			
Alanine amino transaminase, U/L	32.5 ± 3.6	29.6 ± 5.5	0.63
sCr, umol/L	82.3 ± 22.5	90.9 ± 21.6	0.20
eGFR mL/(min·1.73m^2^)	89.4 ± 12.1	85.8 ± 13.6	0.36
LVEF, %	63.0 ± 6.6	61.7 ± 6.1	0.41
White blood cells, × 10^3^/mm^3^	10.5 ± 3.7	11.8 ± 3.7	0.10
Neutrophil, %	77.7 ± 8.6	81.0 ± 8.7	0.08
Hemoglobin, g/dL	137.4 ± 19.9	137.8 ± 16.4	0.91
Platelet counts, × 10^3^/mm^3^	188.3 ± 82.9	154.8 ± 42.4	0.02
PLR	172.3 ± 112.9	169.5 ± 119.2	0.44
NLR	8.5 ± 5.7	9.8 ± 6.2	0.19
Serum fibrinogen level, g/L	3.8 ± 1.7	2.7 ± 1.1	< 0.001
FDP, ug/mL	12.1 (6.3, 28.7)	22.8 (14.9, 43.9)	0.30
D-Dimer, ng/mL	1062 (581, 2650)	2284 (1036, 3145)	0.55
Kidney malperfusion, %	15 (17.9)	10 (11.4)	0.13
Aortic root size, mm	42.1 ± 9.3	38.9 ± 4.7	0.10
Severe aortic regurgitation	34 (40.5)	42 (47.7)	0.50
Ascend aorta size, mm	45.9 ± 7.7	44.8 ± 6.3	0.53
Left ventricular ejection fraction, %	61.7 ± 6.1	63.0 ± 6.6	0.41
*Operation details*			
Bentall + TAR + FET, %	24 (28.6)	32 (36.4)	0.44
Combined with CABG, %	6 (7.1)	6 (6.8)	0.95
The duration of operation, hour	7.6 ± 1.6	8.6 ± 1.6	0.007
CPB time, min	198.2 ± 46.3	225.5 ± 61.2	0.02
Aortic cross clamp time, min	120.5 ± 50.6	130.4 ± 37.6	0.31
The duration of HCA, min	26.9 ± 9.3	28.3 ± 8.1	0.45
Nasopharyngeal temperature, °C	22.5 ± 1.5	23.3 ± 2.2	0.05
Rectal temperature, °C	25.2 ± 2.0	25.7 ± 2.8	0.26
*Postoperative outcomes*			
Length of ICU, day	1.5 (1.0, 3.8)	7.0 (3.6, 13.5)	0.001
Length of hospital, day	14 (10, 20)	16 (12, 19)	0.97
In-hospital mortality, %	2 (2.4)	16 (18.2)	0.02
Reoperation for bleeding, %	2 (2.4)	14 (15.9)	0.03
Postoperative RRT, %	0	38 (43.2)	< 0.001
Multi-organ failure, %	0	18 (20.5)	< 0.001
Sepsis, %	2 (2.4)	28 (31.8)	< 0.001

Results are expressed as *n* (%), mean ± SD or median (interquartile range)

*AKI* acute kidney injury; *ATAAD* acute type A aortic dissection; *BMI* body mass index; *CABG* coronary artery bypass grafting; *CPB* cardiopulmonary bypass; *eGFR* estimated glomerular filtration rate; *FET* frozen elephant trunk; *FDP* fibrinogen degradation products; *HCA* hypothermic circulatory arrest; *ICU* intensive care unit; *LVEF* left ventricular ejection fraction; *NLR* neutrophil to lymphocyte ratio; *PLR* platelet to lymphocyte ratio; *RRT* renal replacement therapy; *sCr* serum creatinine; *TAR* total arch replacement

The incidence of postoperative AKI in our patient population was 51.2% (88/172). Among the preoperative characteristics, Table 1 showed that BMI were higher in AKI group when compared to the non-AKI group (*p* < 0.001). There were no significant differences in preoperative laboratory tests between 2 groups expect for serum fibrinogen level and platelet counts (*p* < 0.001 and *p* = 0.02). Not surprisingly, although no significant differences were observed in the duration of aortic cross-clamp and HCA, patients with AKI required longer operations and CPB time when compared to patients with non-AKI (*p* = 0.007 and *p* = 0.02). Among 172 patients with ATAAD, overall in-hospital mortality was 10.5% (18/172). Among the postoperative characteristics, the postoperative clinical outcome was complicated in patients with AKI, with a higher rate of complications, such as longer ICU stay, overall mortality rate, reoperation for bleeding, sepsis, multi-organ failure and RRT (*p* = 0.001, 0.02, 0.03, < 0.001, < 0.001 and < 0.001, respectively).

### Univariate analysis for risk factors of postoperative AKI

On univariate analysis, preoperative characteristics associated with a higher risk of postoperative AKI included serum fibrinogen level, increasing BMI and platelet counts (*p* < 0.001, *p* = 0.013, *p* = 0.006, respectively). In terms of operative variables, longer operation and CPB time were associated with a higher incidence of postoperative AKI (*p* = 0.018 and *p* = 0.04) (Table 2).

**Table 2:** Univariate analysis of risk factors associated with postoperative AKI in patients with ATAAD

Risk factors	OR	95% CI	*p* value
Preoperative serum fibrinogen level	2.416	1.539–3.794	< 0.001
BMI	1.180	1.036–1.343	0.013
Preoperative platelet counts	0.986	0.976–0.996	0.006
The duration of operation	1.439	1.065–1.943	0.018
CPB time	1.009	1.001–1.019	0.040

*AKI* acute kidney injury; *ATAAD* acute type A aortic dissection; *BMI* body mass index; *CI* confidence interval; *CPB* cardiopulmonary bypass; *OR* odds ratio

### Multivariate logistic regression analysis associated with postoperative AKI

The risk factors for postoperative AKI found using multivariate logistic regression analysis are shown in Table 3. A low preoperative serum fibrinogen level [odds ratio (OR), 1.492; 95% CI, 1.023 to 2.476; *p* = 0.021] and BMI (OR, 1.153; 95% CI, 1.003 to 1.327; *p* = 0.046) were still identified as independent risk factors for postoperative AKI in the multivariate logistic regression analysis.

**Table 3:** Risk factors for postoperative AKI in patients with ATAAD in multivariate logistic regression analysis

Risk factors for postoperative AKI	OR	95% CI	*p* value
Preoperative low serum fibrinogen level	1.492	1.023–2.476	0.021
BMI	1.153	1.003–1.327	0.046

*AKI* acute kidney injury; *ATAAD* acute type A aortic dissection; *BMI* body mass index; *CI* confidence interval; *OR* odds ratio

**Fig. 2:**
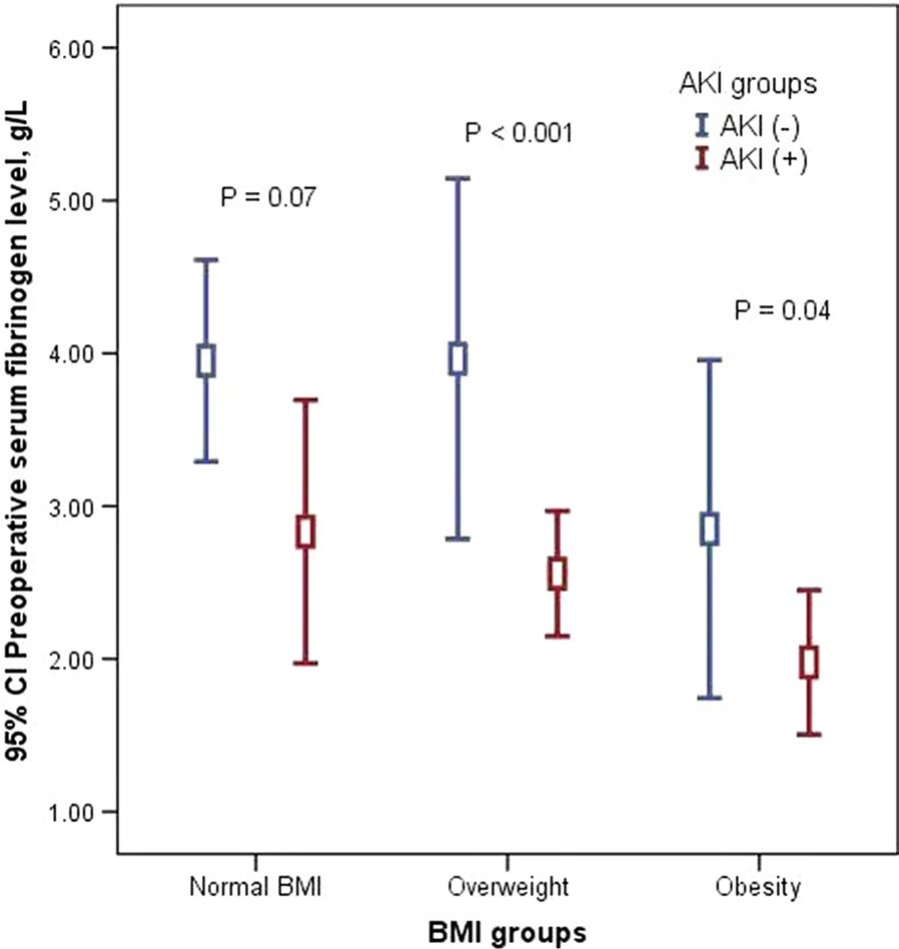
Changes in preoperative serum fibrinogen level among BMI and AKI groups

To control for the potential independent effects of ATAAD on renal function, a mixed effect analysis of variance modeling was performed (Fig. 2). Similar to the overall analysis, this mixed effect analysis of variance modeling demonstrated obese patients with low preoperative serum fibrinogen level had higher incidence of postoperative AKI compared with normal BMI patients with preoperative higher serum fibrinogen level (*p* = 0.04).

### Risk prediction for postoperative AKI

**Fig. 3:**
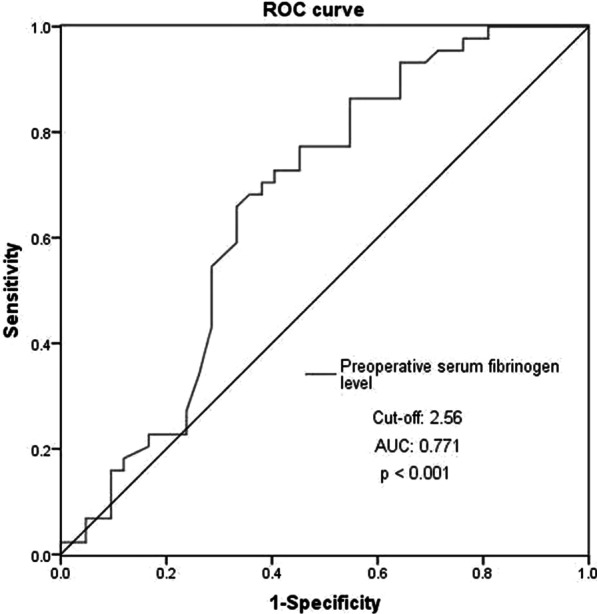
Low preoperative serum fibrinogen level as predictive value of risk factor for postoperative AKI in patients with ATAAD by ROC curve analysis

**Fig. 4:**
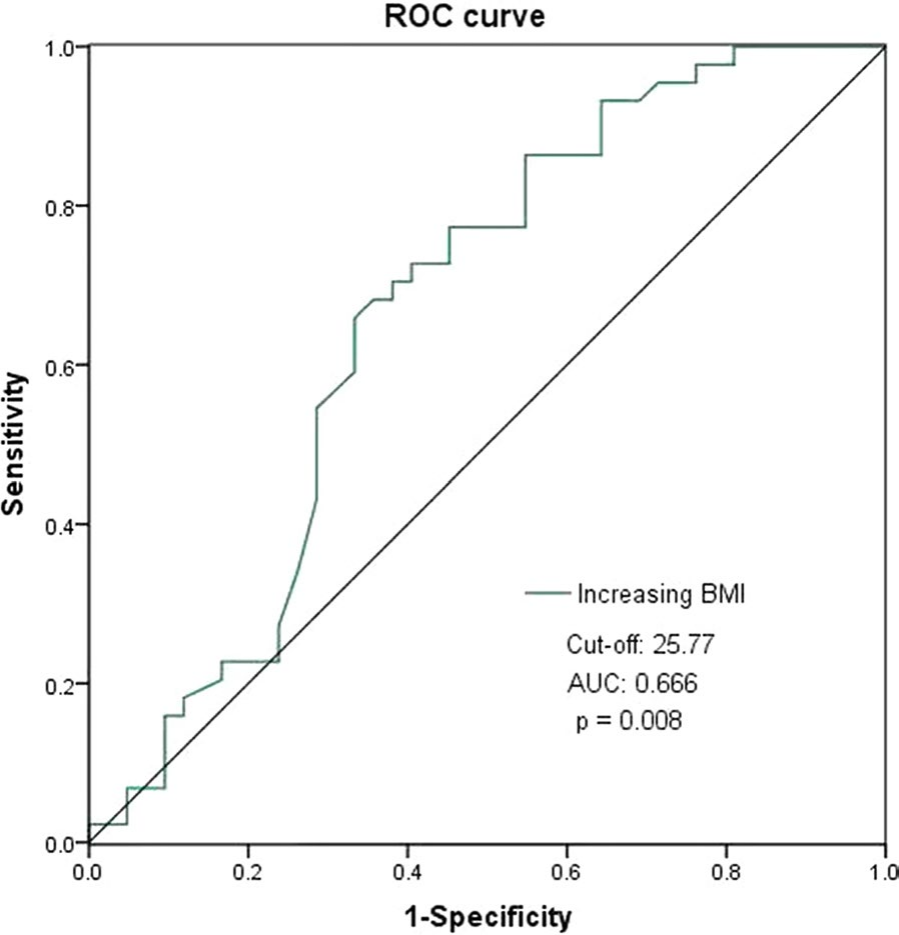
Increasing BMI as predictive value of risk factor for postoperative AKI in patients with ATAAD by ROC curve analysis

The ROC curves were used to determine the predictive and cutoff values of risk factors for AKI. As shown in Figs. 3 and 4, low preoperative serum fibrinogen level (cut-off, 2.56, AUC, 0.771; sensitivity, 85.7%; specificity, 61.4%; *p* < 0.001) and BMI (cut-off, 25.77, AUC, 0.666; sensitivity, 65.9%; specificity, 66.7%; *p* = 0.008) were significant predictors of postoperative AKI.

## Discussion

The main finding of our study was that low preoperative serum fibrinogen level was associated with the risk of developing AKI after TAR combined with a FET, especially in obese patients. In multivariate logistic regression analysis, a low preoperative serum fibrinogen level (< 2.56 g/L) was associated with the risk of developing AKI. The risk was about 1.49 fold higher in patients with low preoperative serum fibrinogen level than in those with a normal or higher fibrinogen level.

As indicated by many studies [3, 12, 13], postoperative AKI has been recognized as a more frequent serious complication in aortic surgery. Vekstein AM et al. [14] and other studies [3, 4, 13] documented the incidence of AKI following aortic surgery of 40%-60%, which was comparable to our finds. Considering that our study cohort underwent emergent aortic TAR combined with a FET implant as a result of ATAAD, the incidence of postoperative AKI (51.2%) was not surprising. Consistent with previous some studies [3, 4, 13–15], patients with AKI in the present analysis had more postoperative complications, such as prolonged hospital or ICU stays, and a higher risk of perioperative mortality than patients with normal kidney function.

Numerous studies aimed to identify risk factors of postoperative AKI in cardiac surgery and some predictors [16–18] have been reported such as advanced age, higher BMI, female, smoking history, hypertension, coronary artery disease, chronic obstructive pulmonary disease (COPD), diabetes mellitus, congestive heart failure, anemia, baseline kidney function, liver disease, hypotension, intra-aortic balloon pump, operation time, CPB time, cross-clamp time, hemodilution, transfusion load, hypothermia, and preoperative exposure to angiotensin converting enzyme inhibitor/angiotensin receptor blocker (ACEI/ARB). Nevertheless, despite the high prevalence, the underlying mechanisms of postoperative AKI after surgery for ATAAD remains unclear. In recent years, there have been considerable advances in our understanding of AKI. The pathophysiology of AKI is associated with hemodynamic perturbations, inflammatory, immunity, iron metabolism, free hemoglobin, increased oxidative stress and associated inflammation [17].

Systemic inflammation is the underlying pathophysiological mechanism of surgery-associated AKI [19, 20]. In addition, fibrinogen is playing an essential role not only in hemostasis and coagulation, but in inflammation, tissue injury, cell migration and cell expression [8]. A few studies have found that serum fibrinogen level was associated with cardiovascular risk factors or cardiovascular events [21, 22]. Celik et al. [23] and Hoffmann et al. [24] reported that an elevated serum fibrinogen level was associated with the occurrence and development of postoperative AKI in patients undergoing percutaneous coronary intervention and abdominal aortic aneurysm repair.

In cardiac surgery, for managing postoperative bleeding, fibrinogen concentrate and its related products is conventionally used as the standard replacement in many European countries [6, 7, 25]. However, several studies [26, 27] had found that a high preoperative fibrinogen level in patients undergoing cardiac valve replacement surgery or heart transplant was a risk factor for postoperative development of AKI. Elevated fibrinogen level is associated with increased blood viscosity, which may increase shear stress and damage endothelial function [28]. The increase in blood viscosity induced by fibrinogen will lead to renal tubular hypoperfusion and hypoxia, which cause AKI [29].

However, Jaesik Park et al. [30] found that the probability of postoperative AKI was two-fold higher in the low fibrinogen group (< 160 mg/dL) than in the normal fibrinogen group in 676 patients who have undergone living donor liver transplantation. Our result was consistent with this, patients in the current study with low preoperative serum fibrinogen level seemed to be more vulnerable to AKI. So far, few studies investigated the relationship between preoperative serum fibrinogen level and the development of postoperative AKI in patients with ATAAD.

In our studies, majority of patients with ATAAD exhibited lower serum fibrinogen level than other cardiac surgery, because ATAAD itself led to a systemic activation of coagulation, possibly resulting in active consumption coagulopathy, such as massive thrombin generation and consumption of clotting factors, fibrinogen or platelets [31]. As fibrinogen was impaired and consumed in false lumen frequently observed in ATAAD, the preoperative serum fibrinogen level often decreased, which could increase the possibility of the development of postoperative AKI. Therefore, unlike patients in previous studies in which AKI was related to high fibrinogen level [26, 27], in patients with ATAAD, low preoperative serum fibrinogen level might be a risk factor for postoperative AKI. Our results suggested that patients with low preoperative serum fibrinogen level were more likely to develop AKI, because ATAAD might be related to fibrinogen consumption, which resulted in an inappropriate response to inflammation. Moreover, low preoperative serum fibrinogen level may be an early and easily measurable surrogate marker for the risk of postoperative AKI in patients with ATAAD. Further understanding of the mechanism of this association is crucial to the design of preventative strategies.

The prevalence of obesity in Western countries is increasing. In the USA, one third of the population is obese, and two thirds are overweight [32]. It is well-known that obesity is a multifactorial disorder which is frequently accompanied by serious co-morbidity and complications, including risk of severe cardiovascular diseases, respiratory diseases, AKI and all causes [5, 33, 34]. Obesity have been reported as predictors for AKI in critical illness populations [35, 36]. Mechanisms proposed for the obesity-AKI link include subclinical chronic kidney disease, intraabdominal hypertension, and alteration in baseline and evoked circulating inflammatory mediators and adipokines [35, 36]. Consistently, we also found that obese patients are more likely to develop postoperative AKI. In the present study, increasing BMI showed a significant influence for postoperative AKI in univariate analysis and multivariate regression model.

### Study limitations

Several potential limitations of the present study should be discussed. First, the patient population collected for investigation was relatively small and only in a single institution, with a relatively young age, and only included the type of aortic TAR combined with a FET implant surgery, which limited the applicability of our findings to other settings. Second, we were unable to identify the mechanisms underlying the association between low fibrinogen level and the development of AKI. Because fibrinogen may not simply reflect coagulation, but it plays a key role in the acute phase response to multi-tissue damage. Therefore, inflammation, coagulation and fibrinolysis may be associated with the development of AKI. However, it is difficult to determine whether those processes are responsible for the development of AKI. Further studies are required to confirm the predictive value of the fibrinogen level for AKI in ATAAD patients. Third, despite the multivariate analysis, some potential bias could have remained due to the retrospective study design. Finally, additional prospective multicenter studies addressing larger sample sizes and experimental studies are necessary to verify our results.

## Conclusions

In conclusion, we found that low preoperative serum fibrinogen level was significantly associated with the risk of AKI in patients with ATAAD in the present study. The results suggested that low preoperative serum fibrinogen level was preferred marker for predicting the postoperative AKI, especially in obese patients with ATAAD. Thus, we recommended that sufficient supplementation of fibrinogen concentrate might improve coagulopathy and decrease the risk of AKI in patients with ATAAD.

## Data Availability

The datasets used or analysed during the current study are available from the corresponding author on reasonable request.

## Abbreviations

ACEI: Angiotensin converting enzyme inhibitor
AKI: Acute kidney injury
ARB: Angiotensin receptor blocker
ATAAD: Acute type A aortic dissection
AUC: Area under the curve
BMI: Body mass index
CI: Confidence interval
COPD: Chronic obstructive pulmonary disease
CPB: Cardiopulmonary bypass
eGFR: Estimated glomerular filtration rate
FET: Frozen elephant trunk
HCA: Hypothermic circulatory arrest
ICU: Intensive care unit
KDIGO: Kidney Disease Improving Global Outcomes
OR: Odds ratio
ROC: Receiver operator characteristic
RRT: Renal replacement therapy
sCr: Serum creatinine
SD: Standard deviation
TAR: Total arch replacement
